# Ocular abnormalities in patients with beta thalassemia on transfusion and chelation therapy: Our experience

**DOI:** 10.4103/0301-4738.67061

**Published:** 2010

**Authors:** Jitendra Jethani, Kenshuk Marwah, Nikul, Shashank Patel, Bela Shah

**Affiliations:** Dr. Thakorbhai V Patel Eye Institute, Vadodara, India; 1 M & J Western Regional Institute of Ophthalmology, Ahmedabad, India; 2 Department of Pediatrics, BJ Medical College, Ahmedabad, India

Dear Editor,

We read with interest the article by Taneja *et al*.[1] We believe that it was a great effort considering the number of studies on this particular group and no such study in our population group. There is relatively low awareness about these ophthalmic manifestations of thalassemia and this article would go a long way to create awareness about the same.

We want to share our own experience (presented as a free paper in AIOC 2009, Jaipur) which is different from their findings, especially in the untreated and treated group with desferioxamine. A total of 112 children (224 eyes) with beta thalassemia were taken for the study. Ninety-five children (190 eyes, 84.8%) not on desferioxamine therapy were termed as case group and 17 children (34 eyes, 15.2%) were on desferioxamine and called control group. Out of 95 children, 62 (65.2%) were male. All children were under the age of 15 years (4-15 years) who had received multiple blood transfusions.

Conjunctival blanching and isolated cataractous changes in the lens were the most common anterior segment findings in the untreated group. Both were found in 12 (6.3%) eyes. The rest of the findings are listed in Table 1. In the group on desferioxamine, none of these children had any opacity in the lens. Four (11.8%) had tessellated fundus in the control group.

**Table 1:** Various ocular manifestations in the untreated group

Anterior segment findings	No. of eyes (%)	Posterior segment findings	No. of eyes (%)
Conjunctival Blanching	12 (6.3)	Tessellated fundus	55 (28.9)
Lens opacities	12 (6.3)	Temporal pallor	8 (4.2)
Xerosis/ Bitot’s spot	8 (4.2)	Area of pigmentation	6 (3.1)
Heterochromia	4 (2.1)	Albinotic fundus	4 (2.1)
Pseduophakia	2 (1)	Nevus	2 (1)
Abduction deficit (lateral rectus underaction)	2 (1)	Venous dilatation	2 (1)
Telecanthus	2 (1)	Nasal tilting	1 (0.5)
Persistent	1 (0.5)	Snow flake degeneration	1 (0.5)
Pupillary membrane		Area of depigmentation	1 (0.5)
		Sheathing	1 (0.5)

The ocular manifestations were compared in the untreated group with respect to serum ferritin [Table 2]. We compared our data with other studies and found lens opacities were commoner in our study (14 eyes 7.3%).

**Table 2:** The distribution of various ocular manifestation visa-vis ferritin levels

Serum ferritin		Less than 500	501-999	1000-4999	5000 and above
Anterior segment findings No. of affected eyes (%)	Conjunctival Blanching			12 (6.3)	
	Lens opacities		2 (1)	10 (5)	
	Conjunctival Blanching			12 (6.3)	
	Xerosis/ Bitot’s spot			8 (4.2)	
	Heterochromia Telecanthus			4 (2.1)	
Posterior segment findings No. of affected eyes (%)	Tessellated fundus		3 (1.6)		
	Temporal pallor		2 (1)		
	Area of pigmentation				
	Albinotic fundus	1 (0.5)	1 (0.5)	3 (1.6)	2 (1)
	Nevus	1 (0.5)		1 (0.5)	
	Venous dilatation			1 (0.5)	

Goldberg *et al*.[2] did not find a single case with lens opacity. However, his patients were mainly with sickle cell hemoglobinopathy and not beta thalassemia patients. Degeneration of retinal pigment epithelium (RPE) and tessellated fundus were common in our study as has been noted in a previous study by Gartaganis *et al*.[3]

We could not find a single case with angioid streaks even when our case series was larger. We saw untreated cases and therefore, could see that the effects on the eye were mainly because of the iron overload and not secondary to chelating therapy as has been thought.

We could not find a single case of lens opacities in children (small sample size) with reduced serum ferritin levels. All these children were on chelating therapy. Ocular manifestation in control group was significantly lower and it was comparable to other studies. This suggests that serum ferritin levels and iron load may not be actually responsible for the ocular manifestations of beta thatlessemia. Our study absolves desferioxamine of any role in causing ocular surface disorders. Rather, it is the disease itself which may be responsible for any such disorder. Desferioxamine may be considered safe so far as the eye is concerned. However, since our sample size was small, such results should be interpreted with caution.
